# On the Rate of Interaction of Sodium Borohydride with Platinum (IV) Chloride Complexes in Alkaline Media

**DOI:** 10.3390/ma14113137

**Published:** 2021-06-07

**Authors:** Magdalena Luty-Błocho, Marek Wojnicki, Edit Csapo, Krzysztof Fitzner

**Affiliations:** 1 Faculty of Non-Ferrous Metals, AGH University of Science and Technology, Al. A. Mickiewicza 30, 30-059 Krakow, Poland; marekw@agh.edu.pl (M.W.); fitzner@agh.edu.pl (K.F.); 2 Department of Physical Chemistry and Materials Science, University of Szeged, Rerrich B. Sqr. 1, 6720 Szeged, Hungary; juhaszne.csapo.edit@med.u-szeged.hu; 3 MTA-SZTE Biomimetic Systems Research Group, Department of Medical Chemistry, Faculty of Medicine, University of Szeged, Dóm Sqr. 8, 6720 Szeged, Hungary

**Keywords:** chloride Pt(IV) complex ions, sodium borohydride, chemical reduction, diborane, zero-order reaction

## Abstract

In this work, sodium borohydride was used as a strong reductant of traces of platinum complex ions. The investigations of the kinetics of redox reaction between platinum(IV) chloride complex ions and sodium borohydride were carried out. For the first time, the kinetic experiments were carried out in a basic medium (pH~13), which prevents NaBH_4_ from decomposition and suppresses the release of hydrogen to the environment. The rate constants of Pt(IV) reduction to Pt(II) ions under different temperatures and concentrations of chloride ions conditions were determined. In alkaline solution (pH~13), the values of enthalpy and entropy of activation are 29.6 kJ/mol and –131 J/mol K. It was also found that oxygen dissolved in the solution strongly affects kinetics of the reduction process. Using collected results, the reduction mechanism was suggested. For the first time, the appearance of diborane as an intermediate product during Pt(IV) ions reduction was suggested. Moreover, the influence of oxygen present in the reacting solution on the rate of reduction reaction was also shown.

## 1. Introduction

One of the most important topics for the past two decades has been the recovery of so-called strategic metals (e.g., platinum group metals, rare earths) accompanied with the production of environmentally friendly waste. It is due to the increase of public awareness and in response to environmental derelicts (for example, Directive 2006/21/EC on the management of waste from extractive industries and amending Directive 2004/35/EC), as well as implementation of UE strategy for raw materials (https://eur-lex.europa.eu/legal-content/EN/TXT/?uri=CELEX:52011DC0025 (accessed on 10 May 2021)). These regulations stimulate the demand for new technologies which will allow effective metal recovery and purification of waste solution. Among metals, platinum is important due to many potential applications in catalysis [1], modern chemistry, medicine [2], and engineering. For platinum recovery, several techniques can be used such as extraction [3], leaching [4], sorption [5], precipitation [6], chemical reduction, and biological methods [7]. However, despite the high level of recovery, the methods mentioned above do not guarantee environmentally friendly waste. There are at least two reasons why it happens. The first is directly related to the recovery process itself, which requires the use of hazardous reagents. Such a substance may penetrate into the waste solution as an unreacted reagent or as a hazardous product of the reaction. The second is related to the form of recovered metals. Especially, metals at nanoscale may be dangerous for life and difficult to recover. They are just toxic and difficult to remove simply by filtration.

In this context, we suggest using sodium borohydride as a strong reductant of platinum ions. It is known that NaBH_4_ is hazardous, but as a result of the reaction with metal precursor it can be neutralized to an environmentally ambient form such as boric acid, borax. These compounds are used as food additives [8]. Moreover, the high efficiency of NaBH_4_ requires an application of a small amount of this reductant. The use of sodium borohydride as a reductant allows for a fast reduction of metal ions and nuclei formation. It in turn promotes the formation of small homogeneous nanoparticles (1–3 nm in diameter) which can be used in any field of science [9,10,11]. The high efficiency of sodium borohydride and thus its strong reducing property depends strongly on the pH of the reacting solution. It has been already confirmed in the literature [12] that its reducing activity increases with the increasing concentration of H_3_O^+^ ions in the solution. This fact is related to the rate of decomposition of NaBH_4_ in water, which accelerates visibly below pH = 9.24. In an aqueous solution, NaBH_4_ dissociates according to the reaction:

(1) $${\mathrm{NaBH}}_{4}\overset{}{↔}{\mathrm{Na}}^{+}+{\mathrm{BH}}_{4}^{-}$$

and in the presence of water, ${\mathrm{BH}}_{4}^{-}$ hydrolyzes spontaneously [13]:

(2) $${\mathrm{BH}}_{4}^{-}+4H_{2}O\overset{}{↔}B{\left(\mathrm{OH}\right)}_{4}^{-}+4H_{2}$$

In the course of this reaction, hydrogen evolves rapidly.

Taking into account reactions of hydrolysis of sodium borohydride and the hexachloroplatinate (IV) [13,14,15], the process of reduction reaction of Pt(IV) ions was investigated under alkaline conditions, which assures the presence of ${\mathrm{BH}}_{4}^{-}$ as the reductant in the solution, as well as [PtCl_5_(OH)]^2−^ ions as the metal precursor. Consequently, the reduction process between Pt(IV) ions and NaBH_4_ in alkaline solution (pH ~ 13) in the simplest form can be described by the reaction:

(3) $${\left[{\mathrm{PtCl}}_{5}\left(\mathrm{OH}\right)\right]}^{2-}+{\mathrm{BH}}_{4}^{-}\overset{k_{1}}{\to }\mathrm{products}$$

where *k*_1_ denotes the rate constant of reaction (3).

The possible product of this reaction can be either Pt(II) complex or metallic platinum particles. Many studies indicate that the process of nanoparticles formation is fast. However, nobody measured the rate of the processes preceding it, i.e., the reduction of Pt(IV) to Pt(II) ions and next Pt(II) to Pt(0). Moreover, there is no reliable model which can describe these stages. In order to follow how sodium borohydride behaves during the reaction with Pt(IV) ions, we decided to undertake a detailed study of the first step in the process of platinum particles formation, i.e., reduction of Pt(IV) to Pt(II) ions. Moreover, based on the obtained kinetic data, the possible mechanism of PtNPs formation will be suggested. The choice of the experimental conditions was related to the elimination of possible processes that could run parallel to the reduction of Pt(IV) ions with sodium borohydride.

## 2. Materials and Methods

### 2.1. Chemicals

The base solution of platinum precursor, i.e., 0.076 M solution of H_2_PtCl_6_ was obtained from the metallic platinum with purity 99.99% (Mennica–Metale Szlachetne, Radzymin, Poland). For this purpose, the metallic platinum was dissolved in aqua regia (mixture of 36% HCl and 65% nitric acid in volumetric ratio 3:1, p.a. Chemland, Stargard Szczeciński, Poland) solution, which was dried four times to remove an excess of nitric acid and at the end in hydrochloride acid to keep the acidic pH of the solution. Before every experiment, the fresh solution of Pt(IV) ions was prepared, then the proper volume of base solution was diluted in deionized water. The proper reducer concentration was obtained by dissolving the powder of sodium borohydride (NaBH_4_, 96%, Fluka, Loughborough, UK) into 0.1 M solution of NaOH (p.a. Chemland, Stargard Szczeciński, Poland), which kept the pH alkaline (∼13) and prevented it from a degradation of NaBH_4_ [16,17,18]. In the case of experiments performed in deaerated solutions, the water and sodium hydroxide solutions were purged with nitrogen for 30 min.

### 2.2. Methods

In the experiments, the redox reaction was monitored at a fixed wavelength (262 nm) using the stopped-flow spectrophotometer (Applied Photophysics, Leatherhead, UK) working in the UV-Vis range (from 190–900 nm wavelength) with the 1-cm or 0.2-cm optical path. The spectrum of the [PtCl_6_]^2^¯ solution in 0.05 M NaOH was recorded to establish the fixed wavelength of 262 nm used as a reference to monitor concentration changes. However, due to the stability diagram (Figure S1, Supplementary materials) this reference refers in fact to [PtCl_5_(OH)]^2^¯ species. All kinetic curves, which include about one hundred data points each, were repeated six times to obtain each value of the observed rate constant (*k*_1,obs_).

### 2.3. Dynamic Light Scattering (DLS)

In order to determine the presence of a solid phase in the aqueous solution and particles size, the DLS method was applied (Nano Zeta-S, Malvern, UK).

## 3. Results

### 3.1. Experimental Conditions

The conditions of the conducted experiments are gathered in Table 1.

### 3.2. Spectra of Reagents and Pt(IV) Complex Ions Stability

**Figure 1:**
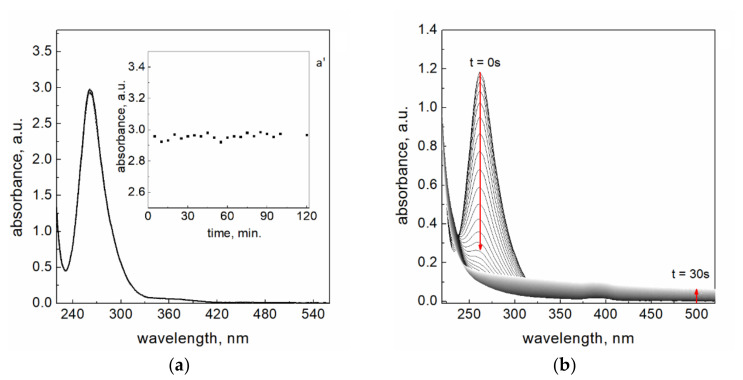
(**a**) Spectrum of aqueous solutions containing 5ˑ10^−4^ M Pt(IV) complex ions in 0.05 M NaOH; kinetic curve for Pt(IV) in 0.05 M NaOH registered at 262 nm (**a’**). Optical path length 2 mm; (**b**) change of absorption bands characteristic for Pt(IV) complex ions during the reaction with NaBH_4_. Conditions: C_0,Pt(IV)_ = 0.05 mM, C_0,NaBH4_ = 3.0 mM, I = 0.05 M, T = 50.0 ± 0.1 °C, pH = 12.9 ± 0.2, path length 1 cm.

The aqueous solution of Pt(IV) complex ions exhibits a strong absorption band in the UV region with maximum at the wavelength (λ) 262 nm (Figure 1a). The localization of the maximum intensity may change depending on the hydrolysis progress (details described by subsequent reactions and stability diagram (Equations (1)–(4), Figure S1, Supplementary Materials). In order to check the spectrum stability, different media were also tested, and the results are given (Figure 1a and Figure S2, Supplementary Materials). It is known, that Pt(IV) complex ions are very stable in comparison to gold(III) and palladium(II) chloride ions. The platinum(IV) complex is more stable in hydrochloric acid and in the solution with the addition of sodium chloride (Figure S2b,c, Supplementary Materials) compared to the water and solution of sodium chlorate (Figure S2a,d,e, Supplementary Materials), which could be expected. For the medium such as water, hydrochloric acid, sodium chloride, sodium chlorate, and sodium hydroxide there is no visible difference between spectra obtained for freshly prepared Pt(IV) solutions. In all these cases, the position of the maximum was located at 262 nm with a small difference in absorbance intensity. However, such results do not prove the presence of the same form of Pt(IV) ions. Murray et al. [18] and Cox et al. [19] observed the same location of spectrum maxima for species such as [PtCl_5_(H_2_O)]¯ and [PtCl_6_]^2−^, and the same value of molar coefficient at 353 nm (ɛ_353nm_ = 490 M^−1^cm^−1^). However, the value of molar coefficient at 262 nm was different and equals 11 600 M^−1^cm^−1^ for [PtCl_5_(H_2_O)]¯ and 24 500 M^−1^cm^−1^ for [PtCl_6_]^2−^. Taking into account the observation by Cox et al. [19], it can be expected that the formed alkaline solution [PtCl_5_(OH)]^2−^ complex (stability diagram in Figure S1b, Supplementary Materials) can also change the value of the molar coefficient. For this purpose, at first, the aqueous solution of [PtCl_6_]^2−^ was obtained by a dissolution of a proper volume of base solution in the deionized water (concentration 0.1 mM) in order to assure [PtCl_5_(H_2_O)]^−^ formation. Then, it was immediately mixed in volumetric ratio (1:1) with 0.1 M solution of NaOH. The obtained spectra were gathered (Figure S3a, Supplementary Materials). Based on them, the value of molar coefficient was determined at 262 nm (ɛ_262nm_ = 29 307 ± 650 M^−1^cm^−1^) (Figure S3b, Supplementary Materials). To be sure, we also checked if the prepared solution reached equilibrium (Equation (3), Supplementary Materials). For this purpose, the spectrum for Pt(IV) ions in 0.05 M NaOH (mixed as previously, i.e., aqueous solution of Pt(IV) ions with 0.1 M NaOH, at volumetric ratio 1:1) were recorded for 2 h (Figure 1a). Small changes in absorbance value in time (Figure 1a’) are related to the fluctuation of measured values. This confirms that the equilibrium has been reached and [PtCl_5_(OH)]^2−^ was formed (Equation (3), Supplementary Materials). It is worth noting that for the experiment in which a proper volume of base solution (0.076 M) of Pt(IV) ions was mixed directly with 10 mL of 0.05 M NaOH, the spectrum with much higher intensity was registered (Figure S4a, Supplementary Materials), and the value of molar coefficient was also established (ε_262nm_ = (34 709 ± 742) M^−1^cm^−1^ (Figure S4b). Moreover, for such a prepared solution, the small decrease in the maximum at 262 nm was observed after 30 min (Figure S5, Supplementary Materials). This result suggests that the equilibrium has not be reached in the considered time or the time needed for conversion of one form into another is much longer (Equation (4), Supplementary Materials). Moreover, what happened with the solution of Pt(IV) complex was tested in different media with time. The UV-Vis spectra registered one month later showed changes in spectrum character, intensity, and maximum location. These changes can be attributed with further hydrolysis process (Figure S2a–e, Supplementary Materials).

Since the reduction reaction of platinum(IV) ions with sodium borohydride takes about 30 s, and overall processes leading to PtNPs formation only 60 s (Figure 1b), we are sure that in the case of hydrolysis reaction of [PtCl_6_]^2−^ and rapid deprotonation of its product, in practice reduction takes place mainly between [PtCl_5_(OH)]^2−^ complex and ${\mathrm{BH}}_{4}{}^{-}$ ions.

**Figure 2:**
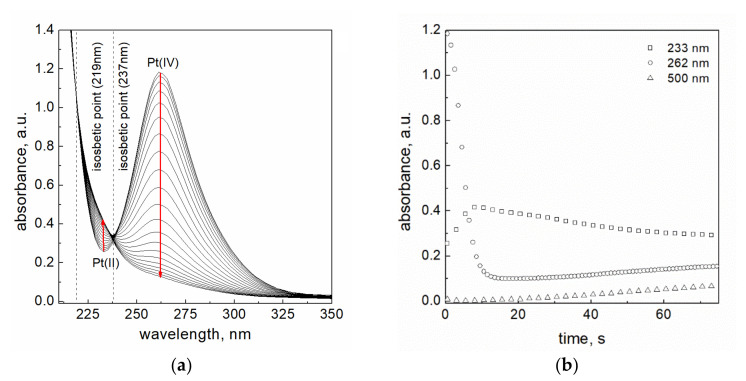
(**a**) Change of absorption bands characteristic for Pt(IV) complex ions during the reaction with NaBH_4_. Conditions: C_0,Pt(IV)_ = 0.05 mM, C_0,NaBH4_ = 3.0 mM, I = 0.05 M, T = 50.0 ± 0.1 °C, pH = 12.9 ± 0.2. Time of reaction 10 s (with step 0.2 s); (**b**) the change of absorbance value (A ∝  concentration of Pt species) with time (kinetic curves) during the reaction of platinum ions with NaBH_4_. Conditions: C_0,Pt(IV)_ = 0.05 mM, C_0,NaBH4_ = 3.0 mM, I = 0.05 M, T = 50.0 ± 0.1 °C, pH = 12.9 ± 0.2, path length 1 cm.

After mixing of the reactants (volume ratio 1:1), the decrease of absorbance for platinum(IV) chloride complex ions in time was observed (Figure 2a). Due to the Lambert-Beer’s Law, this change is due to a decrease of reactant concentration. It shows that the redox reaction (3) between [PtCl_5_(OH)]^2−^ and BH_4_¯ takes place. Moreover, during the reaction between Pt(IV) ions and sodium borohydride, two isosbestic points were detected on the UV-Vis spectrum (Figure 2a). The first one is located at the wavelength 219 ± 1 nm, while the second one at 237 ± 1 nm. A similar value of the wavelength was observed in our previous work [5]. It can be also noted, that with the decreasing maximum value of absorbance at 262 nm (decreasing concentration of Pt(IV) ions), we observed a simultaneously increasing value of absorbance at 233 nm, which can be associated with the formation of Pt(II) ions in the solution. It is compatible with the results of Gammons et al. [20] and Wojnicki et al. [5]. The disappearing peak associated with Pt(IV) ions and the visible change in the absorbance at lower wavelength indicate that the reduction reaction which takes place is:

(4) $${\left[{\mathrm{PtCl}}_{5}\left(\mathrm{OH}\right)\right]}^{2-}+{\mathrm{BH}}_{4}^{-}\overset{k_{1}}{\to }\mathrm{Pt}\left(\mathrm{II}\right)+\mathrm{products}$$

and as a result of this reaction, Pt(IV) ions are reduced to Pt(II) ions.

It is also observed, that after about 4 s, the further decrease of absorbance in the whole considered range was registered. Then, after next 10 s, an increase of absorbance level in the range of wavelength from 250 to 550 nm (Figure 1b) is observed, and it is associated with the appearance in the solution metallic platinum of nanometric size. It was confirmed experimentally with the DLS method, which detected particles of nanometric (4.4 ± 1.4 nm in hydrodynamic radius, Figure S6 Supplementary Materials) size (2 min after mixing of reagents). It is worth noting that the formed particles are unstable and continue to grow up to 44 ± 20 nm (10 min after mixing of reagents) (Figure S7, Supplementary Materials).

Considering the registered absorbance at 262, 233, and 500 nm (value established for the recording of turbidity level in the solution, see details in Figure S8, Supplementary Materials) with time (Figure 2b), we can depict its change for all stages of the reduction reaction. However, it must be underlined that using the spectrophotometric method only the changes of Pt(IV) ions concentration with time can be followed. Nevertheless, one can imagine also the relative changes of concentrations of Pt(II) and Pt(0) with time, which let us divide the whole process into three stages.

Consequently, taking all these facts into account, a possible mechanism of reduction reaction can be suggested:

I-st stage, reduction reaction of Pt(IV) ions to Pt(II) in accordance with the following scheme:

(5) $$\mathrm{Pt}\left(\mathrm{IV}\right)\overset{k_{1,\mathrm{obs}}}{\to }\mathrm{Pt}\left(\mathrm{II}\right)$$

II-nd stage is responsible for the reduction reaction of Pt(II) ions to Pt(0), i.e., it is the nucleation process:

(6) $$\mathrm{Pt}\left(\mathrm{II}\right)\overset{k_{2,\mathrm{obs}}}{\to }\mathrm{Pt}\left(0\right)$$

III-rd stage, describing autocatalytic growth, is typical for noble metals nanoparticles formation [21,22,23,24,25,26,27]:

(7) $$\mathrm{Pt}\left(\mathrm{II}\right)+\mathrm{Pt}\left(0\right)\overset{k_{3,\mathrm{obs}}}{\to }2\mathrm{Pt}\left(0\right)$$

where *k*_1,obs_, *k*_2,obs_, *k*_3,obs_ denote the observed rate constants in Equations (5)–(7).

With the experimental technique used in this work, only the first stage of this suggested mechanism, i.e., the rate of reaction (5) can be investigated.

### 3.3. The Stoichiometry of Reduction Reaction of Pt(IV) Using Sodium Borohydride

The stoichiometry of reduction reaction of Pt(IV) ions using sodium borohydride was determined under a different molar ratio of reagents (Table S1, Supplementary Materials). The concentration change was followed by spectrophotometric observation of the rate of disappearance of the platinum(IV) ions at wavelength 262 nm until its total reduction (absorbance tends to 0). The minimum on the graph (Figure S9, Supplementary Materials) showing the dependence of absorbance on the molar ratio C_0,NaBH4_:C_0,Pt(IV)_ appeared at 2:1 ratio. This means that the reaction of chloride Pt(IV) ions with NaBH_4_ runs in the following proportions:

(8) $${\left[{\mathrm{PtCl}}_{5}\left(\mathrm{OH}\right)\right]}^{2-}+2{\mathrm{BH}}_{4}^{-}\overset{k_{1}}{\to }\mathrm{Pt}\left(\mathrm{II}\right)+\mathrm{products}$$

### 3.4. Determination of the Rate Constants

**Figure 3:**
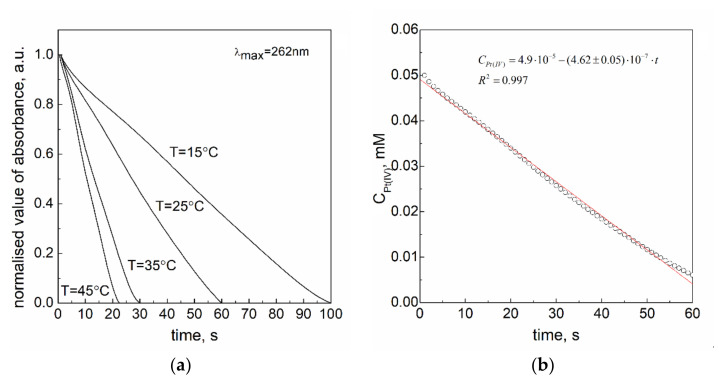
(**a**) The kinetic curves obtained at 262 nm for the reaction between platinum(IV) chloride complex ions and sodium borohydride in aqueous solution at different temperatures; (**b**) the sample of kinetic curve (change of Pt(IV) ions concentration in time of reaction reduction) at 25 °C (**b**). Conditions: C_0,Pt(IV)_ = 0.05 mM, C_0,NaBH4_ = 3.0 mM, pH = 12.9 ± 0.2.

The rate constants of the reduction reaction of Pt(IV) ions with sodium borohydride were established using the equation fitted to the experimental data recorded at the wavelength 262 nm. The obtained kinetic curves registered at different temperatures and the sample of fitted kinetic curve to the obtained data are given in Figure 3.

The character of these curves is linear and it suggests that the process of reduction of metal ions (Equation (5)) is zero-order. For such a process, the differential equation has the form:

(9) $$-\frac{dA_{Pt\left(IV\right)}}{dt}=k_{1,\;obs}$$

Taking into account that the value of absorbance is proportional to Pt(IV) ions concentration (A $\propto C_{Pt\left(IV\right)}$), Equation (9) has the following form:

(10) $$-\frac{d(C_{Pt\left(IV\right)\times \varepsilon_{262nm}\times l)}}{dt}=k_{1,\;obs}$$

where 𝓔_262_ denotes the molar absorption coefficient for Pt(IV) ions (𝓔_262nm_ = 29 307 ± 499 M^−1^cm^−1^ and *l*-path length (*l* = 1 cm).

Under isolation conditions ($C_{NaBH_{4}}\gg C_{Pt\left(IV\right)}$), the reaction between platinum(IV) chloride complex ions and sodium borohydride is assumed to be a pseudo zero-order, where the observed rate constant is in fact expressed as:

(11) $$k_{1,obs}=k_{1}\times C_{NaBH_{4}}$$

Values of *k*_1,obs_ from the experiments and calculated values of *k*_1_ Equation (11) are gathered in Table 2.

### 3.5. Effect of Temperature

For determination of the parameters in the Eyring equation, the values of observed rate constants (*k*_1,obs_) of reaction (5) were determined. The obtained values of *k*_1,obs_, and *k*_1_ (zero-order rate) are gathered in Table 2. The obtained results show that the value of *k*_1_ increases with the increasing temperature. From the linear equation of modified Eyring relationship [28]:

(12) $$T\times \ln\frac{k_{1}}{T}=\left(\ln\frac{k_{B}}{h}+\frac{\Delta S^{\neq }}{R}\right)\times T-\frac{\Delta H^{\neq }}{R}$$

where *k*_1_ is the zero-order rate constant; *T* is the temperature in K; *R* is the gas constant; *k*_B_ is the Boltzmann constant; *h* is Planck’s constant; $\Delta S^{\neq }$ is the entropy of activation; and$\;\Delta H^{\neq }$ is the enthalpy of activation.

**Figure 4:**
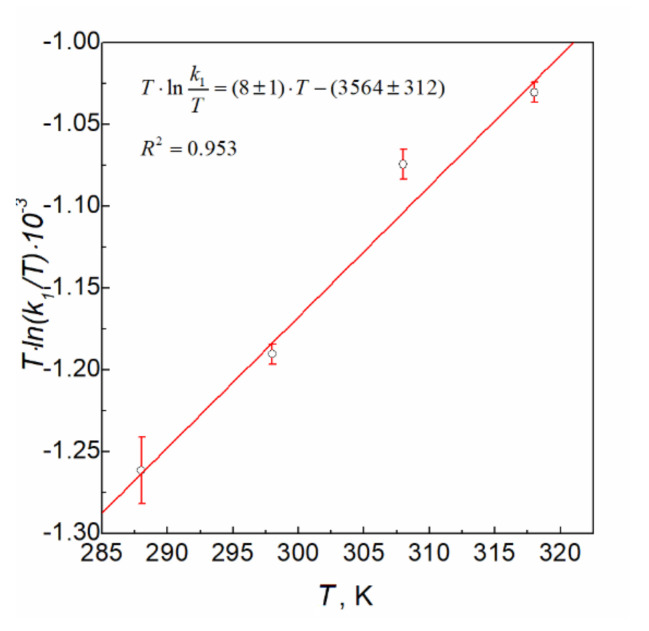
The linear plot of Eyring dependence (*T*·ln(*k*_1_/*T*) vs. *T*) for the reaction between platinum(IV) chloride complex ions and sodium borohydride in aqueous solution. Conditions: C_0,Pt(IV)_ = 0.05 mM, C_0,NaBH4_ = 3.0 mM, pH = 12.9 ± 0.2.

The values of the enthalpy ($\Delta H^{\neq }=29.6\;\mp 2.6\;\mathrm{kJ}\cdot {\mathrm{mol}}^{-1}$) and the entropy ($\Delta S^{\neq }=-131\;J\cdot K^{-1}{\mathrm{mol}}^{-1}$) of activation were calculated by applying the graphical method (Figure 4).

It is worth noting that the process of sodium borohydride decomposition is also dependent on the temperature [13], in which the rise accelerates this reaction. For example, for temperature 288 K, the half time of sodium borohydride decomposition is 283 h, and at 318 K it takes only 27 h [13]. Still, this time is much longer than the time of the experiment and this process can be neglected.

### 3.6. Effect of Chloride Ions

In order to check the influence of chloride ions (Cl¯) addition on the rate constant, different initial concentrations of Cl¯ ions in the solution were used. The obtained results are gathered in Table 3.

**Figure 5:**
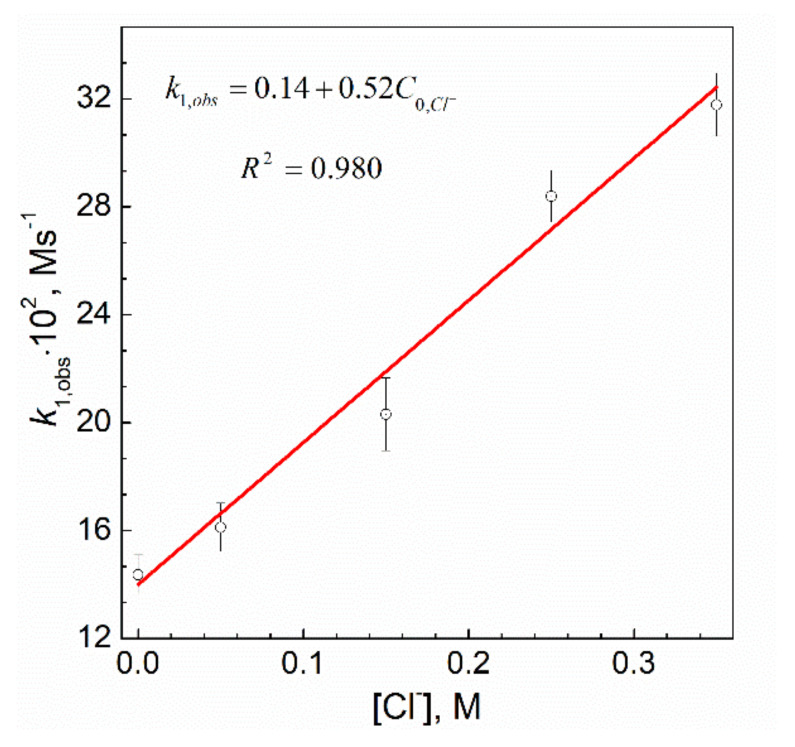
The influence of chloride ions addition on the observed rate constant for the reaction between platinum(IV) ions and sodium borohydride. Conditions: C_0,Pt(IV)_ = 0.05 mM, C_0,NaBH4_ = 3.0 mM, T = 25.0 ± 0.1 °C, pH = 12.9 ± 0.2.

It can be seen that the values of *k*_1,obs_ increase with an increase of Cl¯ ions concentration in the reacting solution. The plot of the observed rate constant vs. the initial Cl¯ concentration is linear and shown in Figure 5.

Fitting the linear equation to these results, the functional dependency of *k*_1,obs_ on the initial concentration of Cl¯ ions takes the following form:

*k_1,obs_* = 0.14 + 0.52*C_0,Cl¯_* (13)

It is worth noting that during the reduction reaction of Pt(IV) ions with sodium borohydride carried out under the conditions described in Table 1 (i.e., at different additions of chloride ions), no visible change in the reacting mixture was observed. In order to explain the observed influence of chloride ions on the reaction rate, two experiments with a much bigger amount of NaCl were carried out. In the first test, the powder of about 0.1 g of sodium chloride was added to the solution with sodium borohydride. In order to enhance the potential effect, the mixture of reductant and salt was not stirred. After a few seconds, at the batch bottom (not diluted salt yet) small bubbles of gas appeared. The second test was performed on the mixture of metal ions with a reductant and addition of NaCl (about 0.1 g). Immediately after the salt addition, the gaseous bubbles appeared in the solution. The intensity of gas evolution was much higher than in the case of the first experiment. These experiments confirmed that the sodium chloride addition enhances the process of sodium borohydride hydrolysis, which is accelerated when metallic platinum is present in the solution. Our results are contrary to those of Gupta et al. [29], who found that the addition of Cl¯ ions to the reaction mixture inhibits the rate.

### 3.7. The Role of Oxygen

**Figure 6:**
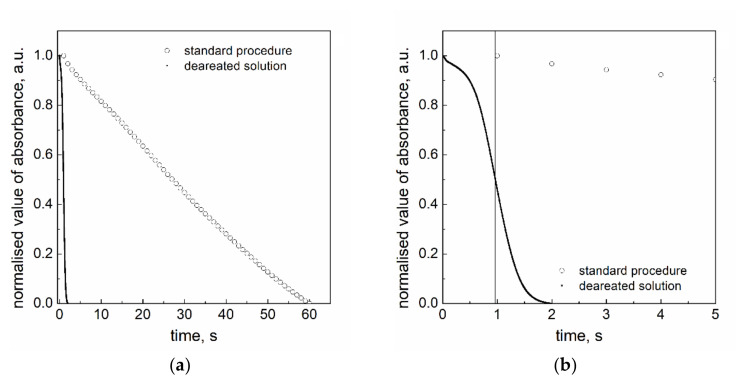
(**a**) Kinetic curves obtained for the reaction between platinum(IV) chloride complex ions and sodium borohydride at a standard procedure and in the case of deaerated solution; (**b**) kinetic curves registered in a short time. Conditions: C_0,Pt(IV)_ = 0.05 mM, C_0,NaBH4_ = 3.0 mM, T = 25.0 ± 0.1 °C, pH = 12.9 ± 0.2.

It is surprising that our studies showed a zero-order reaction for the reduction with sodium borohydride. Therefore, one can think about the reason of such a dependence. However, usually experiments of this type are carried out in the laboratory under air (standard procedure). It is known from the literature, that oxygen plays an important role in the electron-transfer process [30]. A simple molecule can be used as either a catalyst or an inhibitor of the back electron transfer reaction. In order to explain the mechanism of Pt(IV) ions reduction with sodium borohydride for which the obtained kinetic curves are linear, the role of oxygen was studied. For this purpose, the deaerated solution of water and sodium hydroxide was used. The obtained kinetic curves are given in Figure 6.

The character of observed kinetic curves for solutions saturated with oxygen and deaerated one is different. The process runs much faster if oxygen has been removed. The kinetic curve registered for the deaerated solution demonstrates that the Pt(IV) ions reduction consists of at least two steps. The first is slow and the second is fast, maybe also catalytic. Based on this observation, we assumed that oxygen dissolved in the reacting mixture strongly affects the overall process rate. This also confirms that in the case of a standard procedure (solution under air with dissolved oxygen), the oxygen plays the role of the inhibitor.

## 4. Discussion

The sodium borohydride was found to be an efficient reductant of platinum(IV) chloride ions in alkaline (pH about 13) solutions. The reduction reaction of Pt(IV) ions to Pt(II) is fast, and pseudo zero-order. The integral form of kinetic equation describing the concentration change of platinum(IV) complex ions with time have the following form:

(14) $$C_{Pt\left(IV\right)}=C_{0,Pt\left(IV\right)}-\frac{k_{1,obs}}{\varepsilon_{262nm}\times l}\times t$$

Experimental rate constants are sensitive to the temperature. Considering Eyring’s parameters determined in our experiments, the empirical dependence of *k*_1_ = f(*T*) has the following form:

(15) $$k_{1}=\frac{k_{B}\times T}{h}\exp\left(\frac{T\left(-131\;{\mathrm{JK}}^{-1}{\mathrm{mol}}^{-1}\right)-29.6\;{\mathrm{kJmol}}^{-1}}{RT}\right)$$

A negative value for $\Delta S^{\neq }$ indicates that entropy decreases during formation of the transition state. It can result from the associative mechanism in which two reacting species form a single activated complex. Moreover, the addition of chloride ions accelerates the reaction of Pt(IV) ions with sodium borohydride. On the one hand, such a behavior is unexpected taking into account the fact, that the addition of Cl¯ ions affects strongly the stability of the chloride complex. On the other hand, the addition of NaCl to the reductant accelerates the process of NaBH_4_ decomposition. As a result, hydrogen appears, which may also act as a strong reductant.

The reaction is complex and has at least two stages, i.e., the reduction of Pt(IV) ions to Pt(II) and the reduction of Pt(II) to the metallic state Pt(0). As a result of autocatalytic growth (stage III), platinum nanoparticles are formed with a hydrodynamic radius 44 ± 20 nm (10 min after mixing of reagents). The following mechanism of the PtNPs formation can be proposed. Due to hexachloroplatinate (IV) hydrolysis [14] followed by rapid deprotonation of the aqua-complexes, reaction (16) takes place:

(16) $${\left[{\mathrm{PtCl}}_{5}\left(H_{2}0\right)\right]}^{-}\overset{K}{\Leftrightarrow }{\left[{\mathrm{PtCl}}_{5}\left(\mathrm{OH}\right)\right]}^{2-}+H^{+}\to$$

where *K* denotes the equilibrium constant. Under experimental conditions, the equilibrium of this reaction is shifted strongly to the right (Figure S1b, Supplementary Materials). The formed hydrolyzed species can be next reduced in the following chain of reactions:

I stage:

(17) $${\left[{\mathrm{PtCl}}_{5}\left(\mathrm{OH}\right)\right]}^{2-}+2{\mathrm{BH}}_{4}^{-}\overset{k_{1}}{\to }{\left[{\mathrm{PtCl}}_{3}\left(\mathrm{OH}\right)\right]}^{2-}+2{\mathrm{Cl}}^{-}+B_{2}H_{6}+H_{2}$$

II stage:

(18) $${\left[{\mathrm{PtCl}}_{3}\left(\mathrm{OH}\right)\right]}^{2-}+2{\mathrm{BH}}_{4}^{-}\overset{k_{2}}{\to }\mathrm{Pt}+B_{2}H_{6}+H_{2}+3{\mathrm{Cl}}^{-}+{\mathrm{OH}}^{-}$$

III stage:

(19) $${\left[{\mathrm{PtCl}}_{3}\left(\mathrm{OH}\right)\right]}^{2-}+2{\mathrm{BH}}_{4}^{-}+Pt\overset{k_{3}}{\to }2\mathrm{Pt}+B_{2}H_{6}+H_{2}+3{\mathrm{Cl}}^{-}+{\mathrm{OH}}^{-}$$

where *k_i_* (*i* = 1–3) denote the rate constants for reactions (17–19) and consequently reverses the reaction of reduction.

It can be suggested that during the course of reactions (17–19) an intermediated, short-lived product in the form of diborane (B_2_H_6_) is formed. This kind of intermediate was suggested by Pourzahedi and Eckelman [31] during the reduction of silver ions with NaBH_4_. Moreover, Long discussed possible forms of diborane intermediates in alkaline solutions (Long, 1974). In our case it must be unstable [32], and under basic conditions it should react very rapidly with the hydroxyl group according to the following reaction:

(20) $$2B_{2}H_{6}+4{\mathrm{OH}}^{-}\overset{}{\to }3{\mathrm{BH}}_{4}^{-}+{\left[B{\left(\mathrm{OH}\right)}_{4}\right]}^{-}$$

Moreover, the results of this work indicate a huge influence of oxygen dissolved in the solution on the kinetics of redox reaction between Pt(IV) ions and sodium borohydride. The process of metal ions reduction proceeds much faster in the deaerated aqueous solution. Taking into account that oxygen may oxidize the product of reaction (17), it yields:

(21) $$2[{\mathrm{PtCl}}_{3}\left(\mathrm{OH}\right){]}^{2-}+O_{2}+4{\mathrm{Cl}}^{-}+4H_{3}O^{+}\overset{k_{4}}{\to }2[{\mathrm{PtCl}}_{5}\left(\mathrm{OH}\right){]}^{2-}+6H_{2}O$$

where *k_4_* denotes the rate constant in Equation (21).

This reaction pattern can be supported by the following thermodynamic argument.

Taking into account the reaction of Pt(II) with oxygen in the form:

(22) $$2{\left[{\mathrm{PtCl}}_{4}\right]}^{2-}+O_{2}+4{\mathrm{Cl}}^{-}+4H_{3}O^{+}\overset{k_{5}}{\to }2{\left[{\mathrm{PtCl}}_{6}\right]}^{2-}+6H_{2}O$$

where *k_5_* denotes the rate constant in Equation (22), all thermodynamic parameters for this reaction are known [33], and ΔG° (calculated with the Outotec HSC 7 Chemistry software) of this reaction is equal to –192 kJ at 298 K. As it can be seen, this reaction should be spontaneous, and it can be treated as irreversible. One can assume, that reaction (21) exhibits a negative value of ΔG°, and it has to also be spontaneous.

## 5. Conclusions

Summarizing the results of this work one can conclude that:

- The reduction reaction between NaBH_4_ and [PtCl_5_(OH)]^2−^ complex ions can be imagined as a three-stage process:
  (23) $$\mathrm{Pt}\left(\mathrm{IV}\right)\overset{}{\to }\mathrm{Pt}\left(\mathrm{II}\right)\overset{}{\to }\mathrm{Pt}\left(0\right)\overset{}{\to }\mathrm{PtNPs}$$
- Spectrophotometric experiments gave the rate constants of the first stage yielding values of the pseudo-zero order and zero-order rate constants as a function of temperature.
- The obtained values of the enthalpy $\Delta H^{\neq }$ and entropy $\Delta S^{\neq }$ of activation are 29.6 ± 2.6 kJ/mol and −131 J/mol·K, respectively.
- Sodium chloride addition (an increase of Cl¯ ions concentration) enhances the rate of reaction.
- The reduction process is much faster in the deaerated solution. It means that the dissolved oxygen acts as the inhibitor of redox reaction.

## Figures and Tables

**Table 1:** Conditions of experiments for the reaction between platinum(IV) chloride complex ions and sodium borohydride. Conditions: pH = 12.9 ± 0.2, ionic strength I = 0.05 M (except, chloride addition).

Initial Concentration of Reagents, M	Temperature, K	NaCl Addition, M
The stoichiometry of reaction		
*C_0, Pt(IV_*_)_:*C_0,NaBH4_*	–	–
1:2	298	–
1:1	–	–
2:1	–	–
3:1	–	–
–	Effect of temperature	–
0.05 3.0	288	–
–	298	–
–	308	–
–	318	–
Effect of Cl¯ concentration (at constant value of ionic strength I = 0.4 M and [Na^+^] = 0.4 M)		
0.05 3.0	298	0.05
–	–	0.10
–	–	0.20
–	–	0.30
–	–	0.40

**Table 2:** Values of the pseudo zero-order and zero-order rate constants for redox reaction of platinum(IV) chloride complex ions with sodium borohydride obtained at different temperatures. Conditions: C_0,Pt(IV)_ = 0.05 mM, C_0, NaBH4_ = 3.0 mM, pH = 12.9 ± 0.2.

Temperature, K	*k*_1,obs_ 10^3^ Ms^−1^	*k*_1_^.^10 s^−1^
288	10.82 ± 0.18	36.08 ± 0.59
298	13.55 ± 0.07	45.17 ± 0.22
308	28.24 ± 0.24	94.13 ± 0.79
318	37.36 ± 0.22	124.54 ± 0.74

**Table 3:** Values of the observed pseudo-zero- and zero-order rate constants (average of six independent kinetic experiments) for the reaction between platinum(IV) chloride complex ions and sodium borohydride at different Cl¯ concentrations. Conditions: C_0,Pt(IV)_ = 0.05 mM, C_0,NaBH4_ = 3.0 mM, T = 25.0 ± 0.1 °C, pH = 12.9 ± 0.2, [Na^+^] = 0.4 M, I = 0.4 M.

[Cl¯], M	*k*_1,obs_ 10^2^, Ms^−1^	*k*_1_, s^−1^
0.00	14.36 ± 0.73	47.88 ± 2.43
0.05	16.12 ± 0.89	53.74 ± 2.96
0.15	20.30 ± 1.35	67.69 ± 4.50
0.25	28.39 ± 0.96	94.64 ± 3.21
0.35	31.78 ± 1.17	105.93 ± 3.90

## Data Availability

Additional results are contained in Supplementary Material.

## Supplementary Materials

The following are available online at https://www.mdpi.com/article/10.3390/ma14113137/s1. Figure S1: Stability diagram for Pt(IV) complex ions depending on the initial concentration of chloride ions (a) and pH of the solution (b). Conditions: C_0,Pt(IV)_ = 0.05 mM, [Cl¯] = 0.3 mM, T = 25.0 ± 0.1 °C; Figure S2: Spectra of Pt(IV) ions at different media: H2O (a), 0.1 M HCl (b), 0.1 M NaCl (c), 0.1 M NaClO4 (d), NaOH (e). Conditions: C_0,Pt(IV)_ = 0.5 mM, T = 25.0 ± 0.1 °C, path length 2 mm; Figure S3: Spectra of Pt(IV) ions in 0.05 M NaOH (a); dependency of maximum value of absorbance registered at 262 nm vs. initial concentration of Pt(IV) ions (b). Conditions: C_0,Pt(IV)_ = 0.16·10−5 M-0.05 mM, T = 25.0 ± 0.1 °C, path length 1 cm; Figure S4: Spectra of Pt(IV) ions in 0.05 M NaOH (a); dependency of maximum value of absorbance registered at 262 nm vs. initial concentration of Pt(IV) ions (b). Conditions: C_0,Pt(IV)_ = 0.16·10−5 M-0.05 mM, T = 25.0 ± 0.1 °C, path length 1cm; Figure S5: Spectra of Pt(IV) ions in 0.05 M NaOH. Black line–Route I, grey line–Route II. Conditions: C_0,Pt(IV)_ = 0.05 mM, T = 25.0 ± 0.1 °C, path length 1 cm; Figure S6: Size distribution of PtNPs by number (2 min after mixing of reagents). Conditions: C_0,Pt(IV)_ = 0.05 mM, C_0,NaBH4_ = 3.0 mM, T = 25.0 ± 0.1 °C, path length 1 cm; Figure S7: Size distribution of PtNPs by number (10 min after mixing of reagents). Conditions: C_0,Pt(IV)_ = 0.05 mM, C_0,NaBH4_ = 3.0 mM, T = 25.0 ± 0.1 °C, path length 1 cm; Figure S8: The change of absorbance value with time registered at 500 nm. Conditions: C_0,Pt(IV)_ = 0.05 mM, C_0,NaBH4_ = 3.0 mM, I = 0.05 M, T = 25.0 ± 0.1 °C; Figure S9: Graph of the dependence of absorbance as the function C_0,NaBH4_:C_0,Pt(IV)_. Conditions: Different initial concentrations of reagents, pH = 12.9 ± 0.2, T = 298 ± 0.1 K, I = 0.05 M; Table S1: The experimental conditions for determination of reduction reaction stoichiometry.
