# Supplementary material for: On the Rate of Interaction of Sodium Borohydride with Platinum (IV) Chloride Complexes in Alkaline Media
Source: Materials (Basel). 2021 Jun 7;14(11):3137. doi: 10.3390/ma14113137 (PMC8201063; doi:10.3390/ma14113137)
Supplement: Supplementary file 1 [file materials-14-03137-s001.zip › materials-1221135-SI.pdf]

materials MDPI

Supplementary Materials

On the Rate of Interaction of Sodium Borohydride with Platinum (IV) Chloride Complexes in Alkaline Media

Magdalena Luty-Błocho [1,*], Marek Wojnicki [1], Edit Csapo [2,3] and Krzysztof Fitzner [1]

1 Faculty of Non-Ferrous Metals, AGH University of Science and Technology, Al. A. Mickiewicza 30, 30-059 Krakow, Poland; marekw@agh.edu.pl (M.W.); fitzner@agh.edu.pl (K.F.)
2 Department of Physical Chemistry and Materials Science, University of Szeged, Rerrich B. Sqr. 1, 6720 Szeged, Hungary; juhaszne.csapo.edit@med.u-szeged.hu
3 MTA-SZTE Biomimetic Systems Research Group, Department of Medical Chemistry, Faculty of Medicine, University of Szeged, Dóm Sqr. 8, 6720 Szeged, Hungary
* Correspondence: mlb@agh.edu.pl; Tel.: +48-12-617-4126

A. Stability Diagram of $[PtCl_6]^{2-}$

In accordance with literature, in acidic aqueous solution $[PtCl_6]^{2-}$may form mono-aqua and di-aqua complexes in accordance with following equations:

$$[PtCl_6]^{2-} + H_2O \overset{K_1}{\Leftrightarrow} [PtCl_5(H_2O)]^- + Cl^- \tag{1}$$

and $K_1 = \frac{[[PtCl_5(H_2O)]^-][Cl^-]}{[[PtCl_6]^{2-}][H_2O]}$

$$[PtCl_5(H_2O)]^- + H_2O \overset{K_2}{\Leftrightarrow} [PtCl_4(H_2O)_2] + Cl^- \tag{2}$$

and $K_2 = \frac{[[PtCl_4(H_2O)_2]][Cl^-]}{[[PtCl_5(H_2O)]^-][H_2O]}$

and in mild acidic conditions the following species can be present: $[PtCl_{6-n}(H_2O)_n]^{n-2}$ (where n = 0–5) (see, Figure S1).

At higher pH aqua complexes may transform into hydrolyzed form:

$$[PtCl_5(H_2O)]^- \overset{K_3}{\Leftrightarrow} [PtCl_5(OH)]^{2-} + H^+ \tag{3}$$

and $K_3 = \frac{[[PtCl_5(OH)]^{2-}][H^+]}{[[PtCl_5(H_2O)]^-]}$

At pH above 8,

$$[PtCl_6]^{2-} + OH^- \overset{K_4}{\Leftrightarrow} [PtCl_5(OH)]^{2-} + Cl^- \tag{4}$$

and $K_4 = \frac{[[PtCl_5(OH)]^{2-}][Cl^-]}{[[PtCl_6]^{2-}][OH^-]}$

where K_1, K_2, K_3, K_4 the equilibrium constant of reactions (1–4), respectively. For these reactions, values of equilibrium constant equal log K_1 = 1.5–2.2, log K_2 = 3.7 and log K_3 = 3.5. Having initial concentration of $[PtCl_6]^{2-}$, the system of Equations (1)–(4) is solved in Excel, and the resulting equilibrium concentrations are projected on stability diagram (Figure S1).

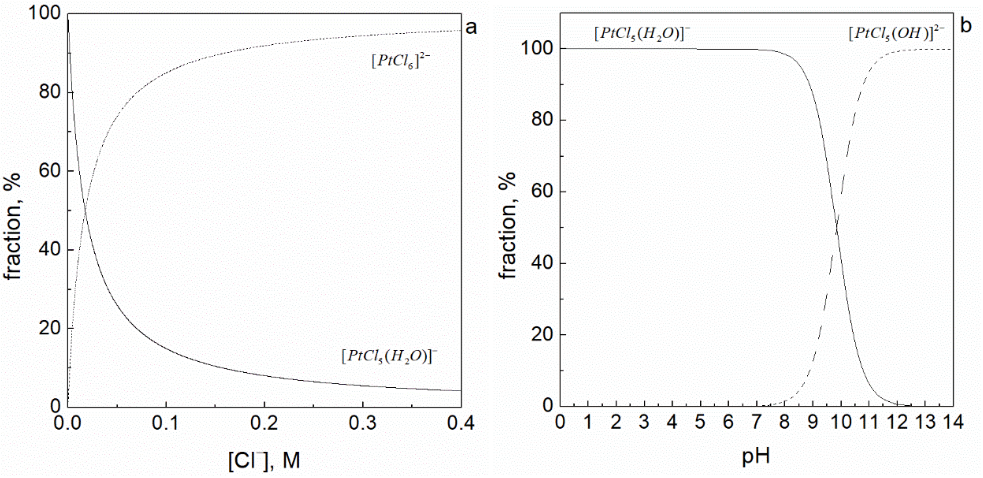

Figure S1. Stability diagram for Pt(IV) complex ions depending on the initial concentration of chloride ions (**a**) and pH of the solution (**b**). Conditions: $C_{0,Pt(IV)}$ = 0.05 mM, [Cl⁻] = 0.3 mM, T = 25.0 ± 0.1 °C.

B. Spectra of Pt(IV) Complex Ions at Different Media

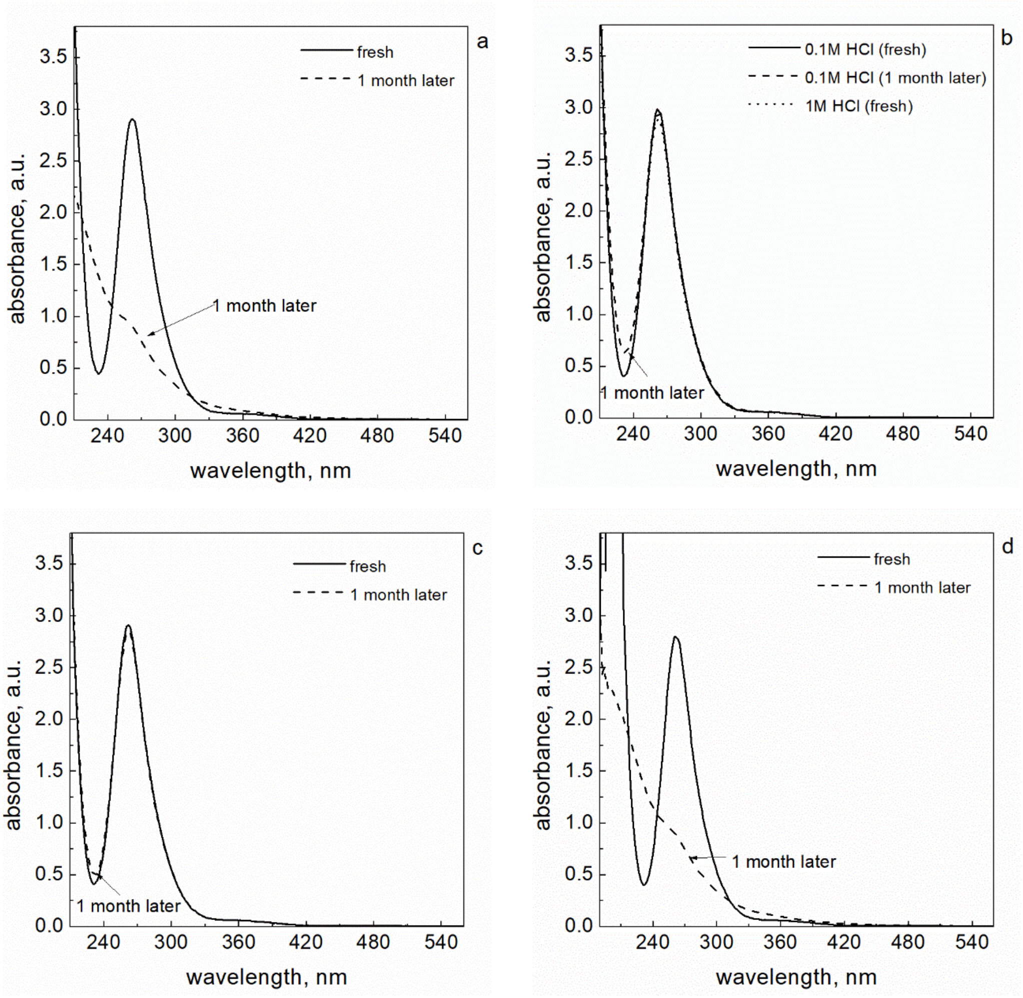

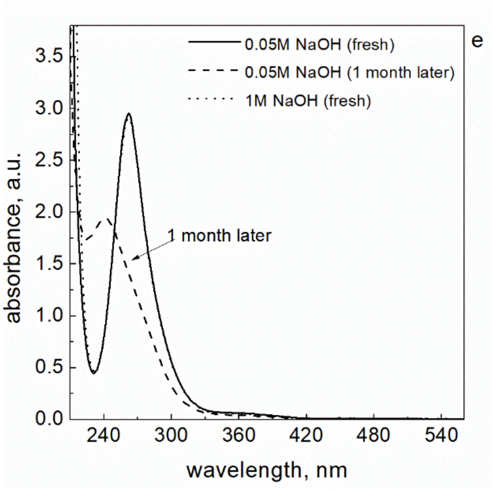

Figure S2. Spectra of Pt(IV) ions at different media: H_2O (**a**), 0.1M HCl (**b**), 0.1 M NaCl (**c**), 0.1 M $NaClO_4$ (**d**), NaOH (**e**). Conditions: $C_{0,Pt(IV)}$ = 0.5 mM, T = 25.0 ± 0.1 °C, path length 2 mm.

C. Spectra of Pt(IV) Complex and Molar Coefficient Determination

Route I: 0.1 mM solution of Pt(IV) ions was prepared by dissolution of proper volume of base solution in 10 mL of deionized H_2O. Next, 1 mL of this solution was mixed in quartz cuvette with 1mL of aqueous solution of 0.1 M NaOH and analyzed. Subsequent dissolution was made by dilution of 1mL of already analyzed mixture with 1 mL of 0.05 M NaOH, etc. Obtained spectra for Pt(IV) ions with concentration in the range form $0.16 \cdot 10^{-5}$ to $5 \cdot 10^{-5}$ M was shown in Figure S3a. According to Lambert-Beer Law, the value of molar coefficient was established from the slope of fitted curve to experimental data point at λ_{max} = 262 nm and it equals ε_{262nm} = (29 307 ± 499) $M^{-1}cm^{-1}$ (Figure S3b).

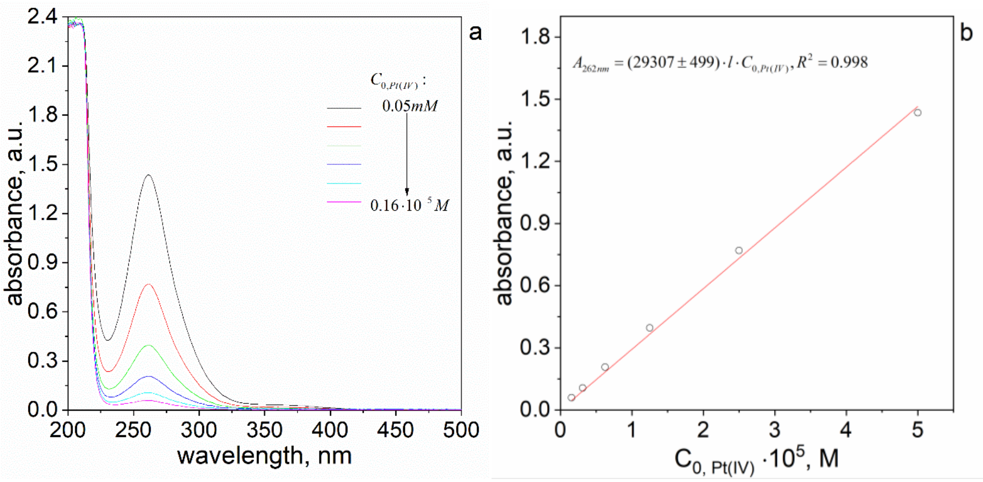

Figure S3. Spectra of Pt(IV) ions in 0.05 M NaOH (**a**); Dependency of maximum value of absorbance registered at 262 nm vs. initial concentration of Pt(IV) ions (**b**). Conditions: $C_{0,Pt(IV)} = 0.16 \cdot 10^{-5}$ M–0.05 mM, T = 25.0 ± 0.1 °C, path length 1 cm.

Route II: 0.05 mM solution of Pt(IV) ions was prepared by dissolution of proper volume of base solution directly in 10 mL of deionized 0.05 M NaOH. Next, quartz cuvette containing 2 mL of the solution was analyzed spectrophotometrically. Subsequent dissolution was made by dilution of 1 mL of already analyzed mixture with 1 mL of 0.05 M NaOH, etc. Obtained spectra for Pt(IV) ions with concentration in the range form $0.16 \cdot 10^{-5}$ to $5 \cdot 10^{-5}$ M was shown in Figure S4a. According to Lambert-Beer Law, the value of molar

coefficient was established from the slope of fitted curve to experimental data point at λ_{max} = 262 nm and it equals ε_{262nm} = (34 709 ± 742) $M^{-1}cm^{-1}$ (Figure S4b).

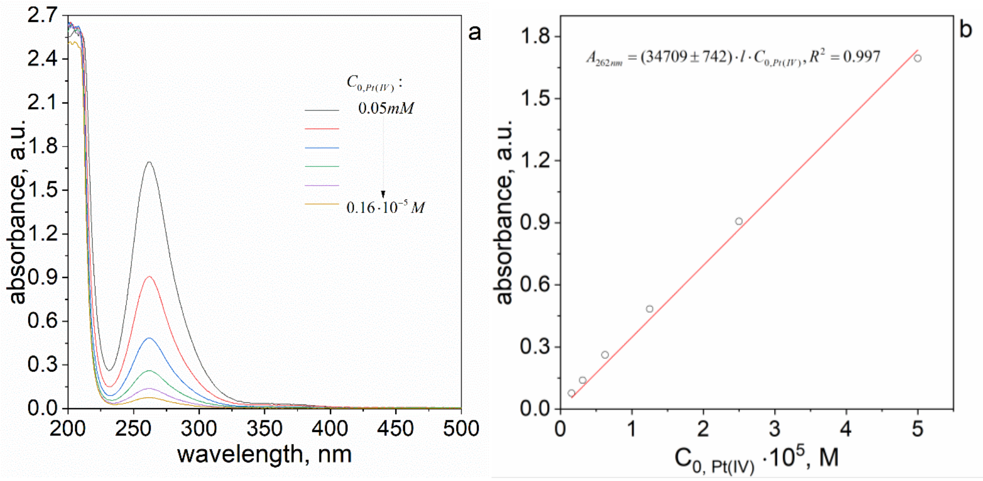

Figure S4. Spectra of Pt(IV) ions in 0.05 M NaOH (**a**); Dependency of maximum value of absorbance registered at 262 nm vs. initial concentration of Pt(IV) ions (**b**). Conditions: $C_{0,Pt(IV)}$ = $0.16 \cdot 10^{-5}$ M–0.05 mM, T = 25.0 ± 0.1 °C, path length 1 cm.

In order to check spectra stability, the solution containing 0.05 mM of Pt(IV) ions obtained in both routes were again analyzed. Obtained results were shown and compared in Figure S5.

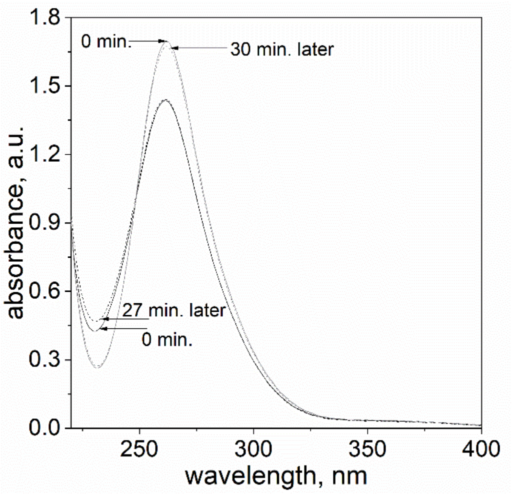

Figure S5. Spectra of Pt(IV) ions in 0.05M NaOH. Black line—Route I, grey line—Route II. Conditions: $C_{0,Pt(IV)}$ = 0.05 mM, T = 25.0 ± 0.1 °C, path length 1 cm.

D. DLS Study

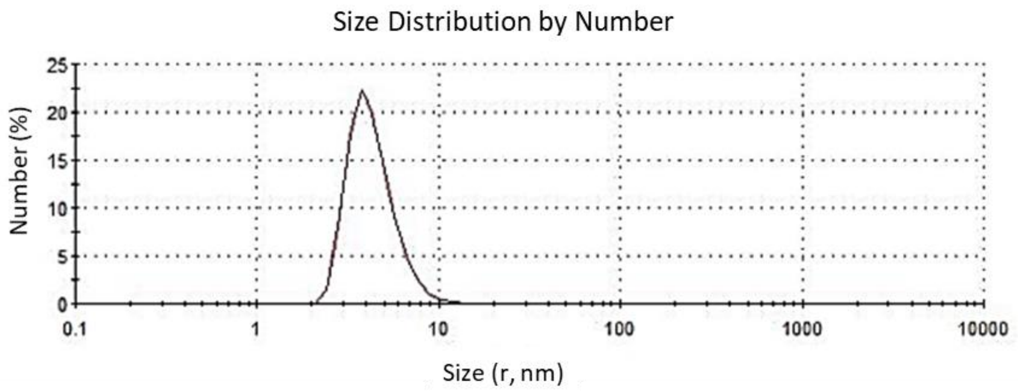

Figure S6. Size distribution of PtNPs by number (2 min after mixing of reagents). Conditions: $C_{0,Pt(IV)}$ = 0.05 mM, $C_{0,NaBH_4}$ = 3.0 mM, T = 25.0 ± 0.1 °C, path length 1 cm.

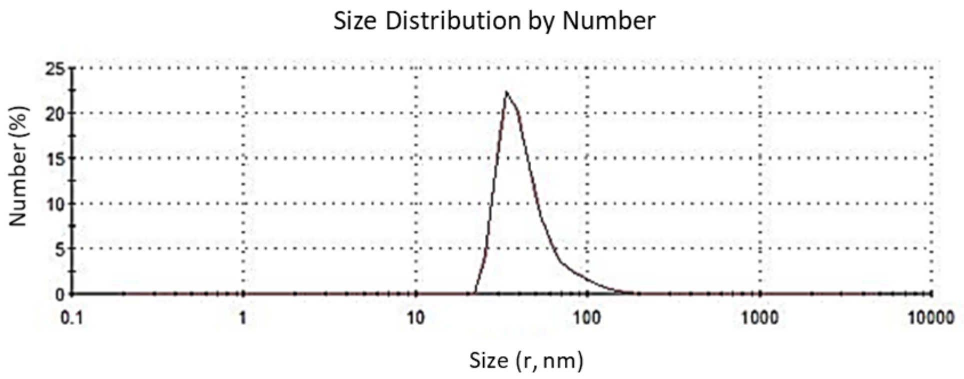

Figure S7. Size distribution of PtNPs by number (10 min after mixing of reagents). Conditions: $C_{0,Pt(IV)}$ = 0.05 mM, $C_{0,NaBH_4}$ = 3.0 mM, T = 25.0 ± 0.1 °C, path length 1 cm.

E. Kinetic Curve Registered for PtNPs

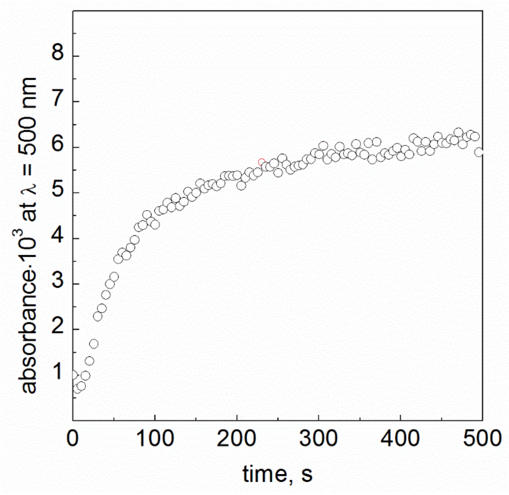

Figure S8. The change of absorbance value with time registered at 500 nm. Conditions: $C_{0,Pt(IV)}$ = 0.05 mM, $C_{0,NaBH_4}$ = 3.0 mM, I = 0.05 M, T = 25.0 ± 0.1 °C.

F. The Stoichiometry of Reduction Reaction of Pt(IV) Using Sodium Borohydride

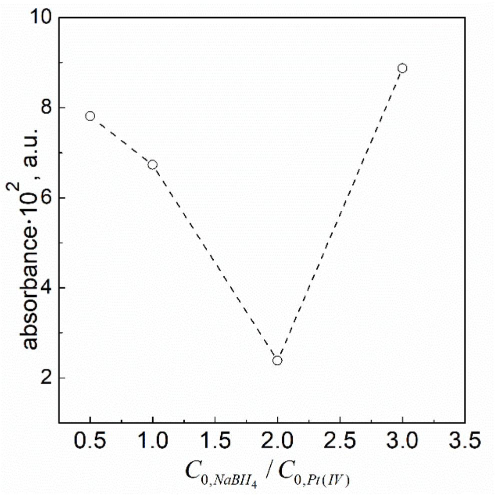

Figure S9. Graph of the dependence of absorbance as the function $C_{0,NaBH4}$:$C_{0,Pt(IV)}$. Conditions: different initial concentrations of reagents, pH = 12.9 ± 0.2, T = 298 ± 0.1 K, I = 0.05 M.

Table S1. The experimental condition for determination of reduction reaction stoichiometry.

The Stoichiometry	Initial Concentration of Pt(IV), mM	Initial Concentration of $NaBH_4$, mM
1:2	0.05	0.10
1:1	0.05	0.05
2:1	0.10	0.05
3:1	0.15	0.05
